# Anticonvulsant use and bone health in a population-based study of men and women: cross-sectional data from the Geelong Osteoporosis Study

**DOI:** 10.1186/s12891-021-04042-w

**Published:** 2021-02-11

**Authors:** Vinoomika Chandrasekaran, Julie A. Pasco, Amanda L. Stuart, Sharon L. Brennan-Olsen, Michael Berk, Jason M. Hodge, Rasika M. Samarasinghe, Lana J. Williams

**Affiliations:** 1 IMPACT, The Institute for Mental and Physical Health and Clinical Translation, School of Medicine, Deakin University, PO Box 281, Barwon Health, Geelong, Vic 3220 Australia; 2 Barwon Health, University Hospital, Geelong, Australia; 3 Department of Medicine-Western Health, The University of Melbourne, St Albans, Australia; 4 Department of Epidemiology and Preventive Medicine, Monash University, Prahran, Australia; 5 Australian Institute for Musculoskeletal Science (AIMSS), The University of Melbourne and Western Health, St Albans, Australia; 6 Deakin University, School of Health and Social Development, Geelong, Waterfront Australia; 7 Institute for Health Transformation, Deakin University, Burwood, Australia; 8 Department of Psychiatry, University of Melbourne, Parkville, Australia; 9 Florey Institute of Neuroscience and Mental Health, Parkville, Australia; 10 Orygen the National Centre of Excellence in Youth Mental Health, Parkville, Australia; 11 Geelong Centre for Emerging Infectious Diseases, Geelong, Australia

**Keywords:** Bone mineral density, Quantitative heel ultrasound, Anticonvulsants, Osteoporosis, psychiatry, neuroscience, medical comorbidity

## Abstract

**Background:**

Anticonvulsant use has been linked to bone deficits in specific patient populations. We studied the association between anticonvulsant use and bone health in a population-based sample of men and women.

**Methods:**

Data from 926 men (24-73 yr) and 1070 women (21-94 yr) participating in the Geelong Osteoporosis Study were included. Bone mineral density (BMD, g/cm^2^) of the PA-spine and total hip was measured using dual-energy X-ray absorptiometry (Lunar). Bone quality was determined using quantitative heel ultrasound (QUS). Anthropometry was conducted and socioeconomic status was determined. Medication and lifestyle information was obtained via questionnaire. Linear regression was used to test associations between anticonvulsant use and bone health before and after adjustment for potential confounders.

**Results:**

Seventeen (1.8%) men and 20 (1.9%) women reported anticonvulsant use. In men, anticonvulsant users had 9.1% lower adjusted mean BMD at the spine and hip compared to non-users. Body mass index was an effect modifier at the spine. Anticonvulsant users also had 1.8% lower speed of sound (SOS), 10.6% lower broadband ultrasound attenuation (BUA) and 13.7% lower stiffness index (SI) compared to non-users. In women, BMD tended to be lower at the hip compared to non-users as with the bone quality measure, BUA. No significant associations were observed at the spine or the other bone quality measures, SOS and SI.

**Conclusion:**

Our data suggest that bone quantity and quality, assessed using BMD and QUS, are lower for men and possibly women who use anticonvulsants. While further exploration into potential mechanisms is needed, our findings suggest that monitoring bone health among users of anticonvulsants is warranted.

## Background

Osteoporosis is an osteodegenerative disease of multifactorial aetiology, often undiagnosed until a fracture occurs [1]. As a growing public health concern, osteoporosis also affects independence, quality of life and increases risk of mortality [2]. The lifetime risk of developing an osteoporotic fracture in industrialised nations is 40–56% for women and 20–30% for men above the age of 50 years [3]. Among those aged 50 years and over, the prevalence of osteoporosis or low bone mass is estimated to increase by 31% between 2012 and 2022 [4].

Dual energy X-ray absorptiometry (DXA) measures bone mineral density (BMD), and is currently the key assessment tool for diagnosing osteoporosis [5]. However, being a 2-dimensional measure, BMD does not sufficiently explain variance in fracture outcomes amongst individuals with similar BMD measures [6], thus additional bone measures may be useful. Quantitative heel ultrasound (QUS) is a radiation-free, portable, non-invasive, inexpensive screening method to assess bone quality, measuring 3-dimensional parameters such as bone structure and elasticity to explain variance in bone strength [5, 7]. Trabecular bone in sites such as the vertebral body and the calcaneus is more susceptible to the effects of altered metabolism [8].

The risk factors for osteoporosis are multifactorial and include age, poor lifestyle, inadequate nutrition, smoking, substance abuse and certain medications and illnesses [1, 9]. Several medications, including anticonvulsants such as valproate, have been associated with increased fracture risk [10]. Anticonvulsants are a mainstay in the treatment of psychiatric and neurological illnesses and have been theorised to have agonistic effects on the glutamatergic and GABAergic neuronal pathways [11] and/or calcium, sodium and potassium voltage gated channels, thereby reversing their neuroexcitatory effects [12]. Other possible mechanisms associated with an anticonvulsant-related decline in bone health are impaired calcium absorption or decreased calcium availability due to calcitonin deficiency, altered sex hormones, liver enzyme induction, inhibition of response to parathyroid hormone or hyperparathyroidism [13, 14]. Further, any potential anticonvulsant-induced alterations in body composition may additionally influence fracture risk [15].

Anticonvulsants have been associated with increased fracture risk in adults [10, 16] and decreased BMD in children [17]. Few studies have investigated anticonvulsant use on QUS parameters [18, 19]. A preclinical analysis of levetiracetam use in rats decreased bone quality, but not BMD, suggesting that DXA measurements may not sufficiently detect all anticonvulsant-related bone deficits [20]. Large studies simultaneously assessing how anticonvulsant use affects bone quality and quantity, after considering potential confounding factors are also sparse. Thus, we aimed to assess the relationship between anticonvulsant use and bone quantity and quality in a large population-based sample of men and women.

## Methods

### Participants

This study utilised data from men and women participating in the Geelong Osteoporosis Study (GOS), a large, ongoing, population-based study conducted in the Barwon Statistical Division in south-eastern Australia [21]. Initially, 1494 women (aged 20–94 years, participation 77.1%) and 1540 men (aged 20–93 years, participation 67%) were randomly selected from the Commonwealth of Australia electoral rolls (where voting is compulsory), between 1994 and 1997, and 2001 and 2006, respectively. Participants have been invited for two to five yearly assessments. An additional sample of 246 women aged 20–29 years was recruited (participation 70.9%) between 2004 and 2008, allowing for continuing investigation of the full adult age range.

For this cross-sectional analysis, data collected at the 10-year follow-up for women and 5-year follow-up for men was utilised. Of the1127 women who participated in the 10-year follow-up, participants for whom bone data were not available were excluded, resulting in a final sample of 1070, aged 20–94 years. Of the 978 men who participated in their 5-year follow-up, similarly, participants for whom bone data were not available were excluded, resulting in a final sample of 926, aged 24–98 years.

### Measurement of the outcome variables

BMD (g/cm^2^) was measured at the spine (PA projection, L2–4) and total hip for men (Lunar Prodigy, GE, Madison, WI, USA) and women (DPX-L, GE, Madison, WI) [21]. Trained technicians carried out all examinations and performed daily calibrations of the densitometers with an equipment-specific phantom.

At the same time as BMD was measured, bone quality was determined by calcaneus QUS (Achilles Insight Ultrasonometer, GE Lunar, Madison, WI, USA) of the left heel, yielding the following parameters: broadband ultrasound attenuation [BUA (dB/MHz)], reflecting microarchitecture and bone density, speed of sound [SOS (m/s)], reflecting elasticity and bone density and stiffness index [SI (%)], a calculated clinical index [22]. SOS and BUA measure the speed and frequency-dependent ultrasound attenuation of ultrasound signals passing through soft tissue and trabecular bone [23]. SI is a combined parameter, calculated from these primary measures [24].

### Measurement of exposure variables

The following data were collected concurrently with the bone health assessments:

#### Medication use

Current medication use was determined via self-report. Participants were requested to bring a medication list or containers to their appointment to ensure accurate reporting. Exposure to anticonvulsants, and other medications known to affect bone, such as oral glucocorticoids, bisphosphonates, and thyroid medication were coded based on the Australian index of medications guidelines.

#### Questionnaire data

Information on daily alcohol use (g/day) and calcium intake (mg/day) was determined using a validated food frequency questionnaire [25]. Participants were classified as smokers if they reported current use at the time of assessment and habitual physical activity level was classified as active if vigorous or light exercise was performed regularly; otherwise participants were classified as sedentary.

#### Other markers of bone health

Osteoporosis status was identified as T-score < − 2.5 at either the spine or total hip [26, 27]. Information regarding previous adult fracture was ascertained via radiological reports from medical imaging centres servicing the region. This method of fracture ascertainment has been previously validated [28].

#### Anthropometric measures

Body mass index (BMI) was calculated (kg/m^2^) from height, measured to the nearest 0.1 cm, and body weight measured to the nearest 0.1 kg*.*

#### Socioeconomic status

Area-based socio-economic status (SES) was established by matching the x-y coordinates of participants’ residential addresses to the Australian Bureau of Statistics’ Index of Relative Socioeconomic Advantage and Disadvantage (IRSAD) data to determine a score for each participant. The IRSAD consists of information regarding income and skill level. IRSAD scores for this study were determined according to cut points of the Barwon Statistical Division and categorised into quintiles whereby SES quintile 1 was considered most disadvantaged and SES quintile 5 was most advantaged.

### Statistical analyses

Minitab (Version 18; Minitab, State College Pa) was used to perform statistical analyses. Differences between anticonvulsant users and non-users were detected using t-tests for continuous normally distributed variables, Kruskal-Wallis for non-parametric continuous variables and chi-square or Fisher’s exact test for discrete variables. Multiple regression was used to explore associations between anticonvulsant use and a) BMD and b) QUS measures (BUA, SOS and SI). Data for men and women were analysed using separate statistical models. Confounders including age, BMI, smoking status, SES, physical activity and medications known to affect bone were tested sequentially and retained in the final model when significant (*p* < 0.05). Interactions between exposure variables were checked for effect modification in the final models.

## Results

### Male sample

Seventeen men (1.8%) were anticonvulsant users at the time of assessment; phenytoin (*n* = 7), carbamazepine (*n* = 5), sodium valproate (n = 5), pregabalin (*n* = 1) and clonazepam (n = 1). Anticonvulsant users were older, less active, consumed less alcohol and were more likely to have a previous adult fracture than non-users; otherwise the groups were similar in regard to BMI, smoking status, calcium intake, osteoporosis status and SES (Table 1). Unadjusted BMD and QUS parameters are shown in Table 1.

**Table 1:** Characteristics of participants, expressed as median (interquartile range), mean (± standard deviation) or n (%)

	Men				Women			
	All	Users	Non-users	p	All	Users	Non-users	p
	***n*** = 926	***n*** = 17	***n*** = 909		***n*** = 1070	***n*** = 20	***n*** = 1050	
Age (yr)	60.0 (46.5–73.2)	66.3 (54.0–84.8)	59.9 (46.2–73.1)	0.05	51.0 (34.7–65.7)	52.3 (34.0–65.1)	51.0 (34.7–65.9)	0.90
BMI (kg/m^2^)	27.2 (24.7–29.6)	27.8 (24.6–34.9)	27.1 (24.7–49.9)	0.93	26.3 (23.4–30.9)	27.5 (22.3–40.3)	26.2 (23.4–55.0)	0.98
Smoking (current)	104 (11.3%)	3 (17.7%)	101 (11.2%)	0.40	153 (14.3%)	3 (15.0%)	150 (14.3%)	0.93
Physical activity (active)	659 (71.5%)	7 (41.2%)	652 (72.0%)	0.005	835 (78.1%)	11 (55.0%)	824 (78.6%)	0.01
Calcium intake (mg/day)	882 (689.0–1136.2)	899 (596.0–1132.5)	880 (689.3–1136.3)	1.000	864 (659.4–1091.0)	976 (785.9–1206.5)	864 (659.0–1089.2)	0.16
Alcohol use (g/day)	12.0 (2.1–28.8)	0.4 (0.0–7.9)	12.6 (2.3–29.4)	< 0.001	2.7 (0.3–12.0)	0.7 (0.0–7.5)	2.8 (0.3–12.2)	0.07
Medication (current)								
Glucocorticoids	10 (1.1%)	0 (0%)	10 (1.1%)	–	16 (1.5%)	1 (5.0%)	15 (1.4%)	0.26
Thyroid-affecting medications	10 (1.1%)	0 (0%)	10 (1.1%)	–	47 (4.4%)	1 (5.0%)	46 (4.4%)	0.60
Agents affecting calcium	15 (1.6%)	0 (0%)	15 (1.7%)	–	40 (3.7%)	3 (15.0%)	37 (3.5%)	0.04
Socioeconomic status				0.67				0.36
Quintile 1 (lowest)	150 (16.2%)	1 (5.9%)	149 (16.4%)		170 (16.0%)	2 (10.0%)	168 (16.1%)	
Quintile 2	186 (20.1%)	4 (23.5%)	182 (20.0%)		224 (21.0%)	2 (10.0%)	222 (21.2%)	
Quintile 3	178 (19.2%)	5 (29.4%)	173 (19.0%)		243 (22.8%)	7 (35.0%)	236 (22.6%)	
Quintile 4	203 (21.9%)	4 (23.5%)	199 (21.9%)		211 (19.8%)	6 (30.0%)	205 (19.6%)	
Quintile 5	209 (22.6%)	3 (17.7%)	206 (22.7%)		218 (20.5%)	3 (15.0%)	215 (20.6%)	
Prior adult fracture	269 (29.1%)	9 (52.9%)	260 (28.6%)	0.03	82 (7.7%)	5 (25.0%)	77 (7.3%)	0.003
BMD (g/cm^2^)								
Spine	1.293 ± 0.198	1.258 ± 0.277	1.294 ± 0.197	0.47	1.209 ± 0.188	1.172 ± 0.223	1.210 ± 0.187	0.39
Hip	0.980 ± 0.137	0.928 ± 0.143	1.066 ± 0.142	< 0.001	0.941 ± 0.166	0.803 ± 0.142	0.853 ± 0.161	0.12
Osteoporosis (current)	29 (8.2%)	3 (25.0%)	26 (7.6%)	0.07	74 (7.0%)	3 (15.0%)	71 (6.8%)	0.16
QUS								
SOS (m/sec)	1572.1 ± 41.4	1540.0 ± 57.0	1572.7 ± 40.9	0.003	1571.1 ± 39.0	1562.9 ± 38.8	1571.2 ± 39.0	0.42
BUA (dB/MHz)	120.4 ± 16.0	106.5 ± 17.6	120.7 ± 15.9	0.001	111.0 ± 16.4	103.9 ± 21.0	111.1 ± 16.3	0.09
SI (%)	99.8 ± 20.5	84.4 ± 26.9	100.1 ± 20.2	0.003	94.7 ± 20.7	91.8 ± 28.5	94.7 ± 20.5	0.57

BMI was an effect modifier in the relationship between anticonvulsant use and BMD at the spine, with the relationship differing for those above and below a BMI of 28.5 kg/m^2^. For example, for users vs non-users, age- and BMI- adjusted mean BMD at the spine for men with a BMI of 25 kg/m^2^ was 1.154 (95% CI 1.039–1.269) vs 1.275 (1.260–1.290) g/cm^2^, a BMI of 27 kg/m^2^ was 1.231 (95% CI 1.138–1.324) vs 1.291 (1.278–1.304) g/cm^2^, a BMI of 29 kg/m^2^ was 1.308 (95% CI 1.204–1.412) vs 1.307 (1.293–1.321) g/cm^2^ and a BMI of 31 kg/m^2^ was 1.385 (95% CI 1.244–1.527) vs 1.323 (1.306–1.340) g/cm^2^ (all *p* = 0.033). At the hip, age- and BMI- adjusted mean BMD was lower among anticonvulsant users compared to non-users [0.887 (95% CI 0.830–0.944) vs 0.976 (0.968–0.984) g/cm^2^, *p* = 0.002]. Smoking, physical activity, alcohol and calcium intake, SES and medications known to affect bone did not contribute to the final models.

After adjustment for age, BMI, smoking, alcohol and calcium intake, anticonvulsant use was associated with lower adjusted mean BUA [107.5 (95% CI 99.4–115.7) vs 120.3 (119.2–121.4) dB/MHz, p = 0.002], SOS [1543.1 (95% CI 1521.8–1564.4) vs 1572.0 (1569.1–1574.9) m/sec, *p* = 0.008] and SI [86.2 (95% CI 76.1–96.2) vs 99.9 (98.6–101.3) %, p = 0.008] compared to non-use. Physical activity, SES and medications known to affect bone did not contribute to the final models.

### Female sample

Twenty (1.9%) women were anticonvulsant users at the time of assessment; carbamazepine (*n* = 6), sodium valproate (*n* = 4), clonazepam (*n* = 5), gabapentin (*n* = 3), lamotrigine (*n* = 1) and pregabalin (n = 1). Anticonvulsant users were less active, more likely to have a previous fracture and were more likely to take agents affecting calcium than non-users; otherwise the groups did not differ in regards to age, BMI, smoking status, calcium intake, alcohol use, SES, osteoporosis status or use of thyroid-affecting hormones and adrenal steroid hormones (Table 1). Unadjusted BMD and QUS parameters for users and non-users are shown in Table 1.

After adjustment for age and BMI, there were no differences detected in BMD at the spine between users and non-users of anticonvulsants [1.164 (95% CI 1.090–1.239) vs 1.200 (1.190–1.211) g/cm^2^, *p* = 0.345], however, anticonvulsant users tended to have lower BMD at the hip compared to non-users [0.876 (0.821–0.931) vs 0.930 (0.922–0.938) units, *p* = 0.058].

After adjustment for age and BMI, anticonvulsant use was not associated with mean SOS [1565.0 (95% CI 1547.8–1582.1) vs 1571.0 (1568.7–1573.3) m/sec, *p* = 0.493] or SI [90.8 (95% CI 82.8–98.9) vs 93.7 (92.6–94.8) %, *p* = 0.484] but anticonvulsant users tended to have lower BUA than non-users [104.5 (95% CI 97.8–111.3) vs 110.7 (109.8–111.6) dB/MHz, *p* = 0.075].

## Discussion

This cross-sectional study investigated associations between anticonvulsant use and bone health in a large population-based sample of men and women. After adjustment for confounders, BMD and QUS parameters were generally lower in men using anticonvulsants compared to non-users. In women, this was not clearly the case, with the difference in BMD and QUS parameters between anticonvulsant users and non-users not reaching statistical significance.

Only two studies have looked at how anticonvulsant use affects QUS measures albeit in children and young adults. A small Italian study (*n* = 164, aged 2–21 years) investigating how anticonvulsants affect DXA and QUS measures in girls with or without Rett Syndrome found that anticonvulsant therapy was associated with lower bone measures, although fracture risk was not elevated [18]. Similarly, a cross-sectional study conducted in Spain found that valproate, but not carbamazepine, phenobarbital, lamotrigine, topiramate, vigabatrine or phenytoin was associated with lower QUS measures in children (*n* = 65, aged 6.5 ± 3.1 years) compared to non-users; despite having 27.3% of their treated group taking two or more anticonvulsants [19]. On the other hand, several studies have looked at associations between anticonvulsant use and BMD [29–32], although most studies have been conducted in patients with epilepsy and in paediatric populations [10]. It has been estimated that over 50% of anticonvulsant users develop bone anomalies [29], and studies conducted over time have shown that long-term use is associated with increased fracture risk and associated bone disease [30, 32]. In a large, multiethnic, postmenopausal cohort (*n* = 138,667, aged 50–79 years), anticonvulsant use was associated with an increased fracture risk, which was further increased with polypharmacy and enzyme-inducing anticonvulsants rather than the non-enzyme inducing type [30]. Interestingly, anticonvulsant use was not associated with BMD at the spine, hip and total body, but was associated with falls risk. While our results reflect the association between anticonvulsants and bone health in adults, it appears that our findings are concordant with the existing literature, that while anticonvulsant use may be broadly associated with bone deficits, associations in girls and women are less clear.

Chronic anticonvulsant use has been associated with a 1.2–2.4-fold increase in fracture risk [33]; explained in part by hepatic enzyme-inducing anticonvulsants known to increase conversion of 25-hydroxyvitamin D into inactive metabolites [34]. Mezuk et al. [35] observed that although enzyme-inducing anticonvulsants may pose a greater fracture risk (HR 2.19, 95% CI: 1.97–2.43), non-enzyme-inducing anticonvulsants are also associated with an increased fracture risk (HR 1.66, 95% CI: 1.54–1.79). Additionally, Lee et al. [33] observed that first generation anticonvulsants such as valproic acid, carbamazepine, phenytoin and phenobarbital are associated with an increased rate of fragility fractures, when compared to newer agents.

Confounding by indication is another factor possibly playing a role. Anticonvulsants are used in the treatment of epilepsy and bipolar disorder, with both having been shown to be independently associated with increased fracture risk. In a recent systematic review, bipolar disorder was associated with a 20–80% increased risk of fracture, independent of age, sex, medication and comorbidities [9]. Decreased physical activity and other modifiable risk factors, such as diet, substance use, smoking, SES, sun exposure, medical comorbidities, polypharmacy and drug-induced metabolic imbalances may also contribute to the overall decline in bone health [36–38]. Falling, associated with seizures in epilepsy, has been proposed to be another mediating factor [39].

The results of this study, when taken in context of research showing that anticonvulsant use independently increases fracture risk, add to existing clinical findings, and may assist treatment decisions in managing an already vulnerable population. Adequate calcium supplementation could counteract the deleterious effects of anticonvulsants [40], while clinical trial evidence suggests that the association between calcium supplementation and improved bone health is weak [41]. The antiepileptic drug and osteoporosis prevention trial (ADOPT) reported similar findings, where over 69% of their cohort (*n* = 80 men, aged ≥58 years) had improved BMD measures with adequate calcium supplementation [42].

Both strengths and limitations need to be taken into consideration. Strengths of this study include the representative nature of the sample spanning the full adult age range, and the number of confounding variables tested. A limitation of this study is its cross-sectional nature, preventing conclusions on how BMD and QUS measures varied over time. Second, vitamin D status has not been considered. Other factors for consideration are a likely healthy participant bias and the small number of anticonvulsant users is an additional consideration, which also limited subgroup analyses of specific agents. Lastly, any unidentified confounding may affect our findings.

## Conclusion

Both DXA and QUS measures in our study suggest an anticonvulsant-associated deficit in bone health, at least for men. Further exploration into mechanisms are warranted, as this information may inform clinical decisions and enable necessary preventative measures to be taken when prescribing anticonvulsants.

## Data Availability

Participants’ data available on request from Professor Julie Pasco, due to privacy/ethical restrictions.

## Abbreviations

BMD: Bone mineral density
DXA: Dual-energy X-ray absorptiometry
QUS: Quantitative heel ultrasound
SOS: Speed of sound
BUA: Broadband ultrasound attenuation
SI: Stiffness index
GOS: Geelong Osteoporosis Study
IRSAD: Index of Relative Socioeconomic Advantage and Disadvantage
BMI: Body mass index
HR: Hazard ratio
CI: Confidence interval
SES: Socioeconomic status
ADOPT: Antiepileptic drug and osteoporosis prevention trial
